# 

**Published:** 1876-06

**Authors:** 


### Contents of June Number.

### ORIGINAL.

ART. I.—Inaugural Address of the President of the Medical Society of the State of New York, Dr. Thomas F. Rochester, delivered in Albany, June 20th, 1876, ... 400
ART. II.—Hemorrhage following Miscarriage, and its treatment by Intra-uterine injections. By W. W. Potter, M. D., Mt. Morris, N. Y....... 414
ART. III.—Surgical Cases. By C. C. F. Gay, M. D., ... 416
ART. IV.—Abstract of the Proceedings of the Buffalo Medical Association,.. 420

### MISCELLANEOUS.

Proceedings of the Association of Representatives of American Medical Colleges, ... 424
Twenty-Seventh Annual Meeting of the American Medical Association,.... 429

### EDITORIAL DEPARTMENT.

The American Medical Association, ... 435
Meeting of the Representatives of American Medical Colleges, ... 437
Meeting of the New York State Medical Society, ... 438

Books Reviewed:
A Manuel of Pathology. For the use of Students and Practitioners of Medicine. By Ernst Wagner, ... 439
Books and Pamphlets Received, ... 440

